# Comparative analysis of bone density measurement techniques: a systematic review of quantitative ultrasound and dual-energy X-ray absorptiometry

**DOI:** 10.3389/fendo.2026.1768327

**Published:** 2026-03-04

**Authors:** Ibrahim Hadadi

**Affiliations:** Department of Radiological Sciences, College of Applied Medical Sciences, King Khalid University, Abha, Saudi Arabia

**Keywords:** bone mineral content, bone mineral density, bone quality, DXA, fractures, osteoporosis, QUS

## Abstract

**Background:**

Bone mineral density (BMD) and bone-related parameters are essential for osteoporosis detection. Different screening modalities are used, including quantitative ultrasound (QUS) and dual-energy X-ray absorptiometry (DXA). This systematic review aimed to evaluate the correlation and clinical utility of DXA and QUS measurements.

**Methods:**

A literature search (2005–2025) was conducted in PubMed, Scopus, Web of Science, EMBASE, Google Scholar, and Cochrane Library for English-language studies. A narrative synthesis was performed to summarize the study characteristics and outcomes.

**Results:**

Of the 1,247 identified records, 24 studies met the inclusion criteria. DXA and QUS were used to assess bone parameters, such as BMD, bone mineral content (BMC), speed of sound (SOS), broadband ultrasound attenuation (BAU), and stiffness index (SI). The correlation between DXA and QUS varied widely (r = 0.17–0.86), with variable diagnostic performance across studies. Studies involving postmenopausal women and older populations reported similar trends, whereas the findings were inconsistent in pediatric and disease-specific populations.

**Conclusion:**

QUS is suitable for preliminary screening, especially in resource-limited settings, but cannot replace DXA for definitive diagnosis. Further well-designed studies with longer follow-up are required to better define the role of QUS in osteoporosis screening.

**Systematic Review Registration:**

https://www.crd.york.ac.uk/prospero/, identifier CRD420251146250.

## 1 Introduction

Osteoporosis is characterized by a progressive decline in bone mineral density (BMD) and deterioration of the bone microarchitecture, which substantially increases skeletal fragility (1, 2). Often remaining asymptomatic until the occurrence of a fragility fracture, the condition is frequently underdiagnosed, despite its significant global health impact. Such fractures account for millions of new clinical cases annually and are a primary driver of increased morbidity and mortality in aging populations (3–5).

Although the World Health Organization (WHO) diagnostic criteria for osteoporosis remain centered on BMD T-scores measured at clinically relevant skeletal sites, the clinical application of these standards relies heavily on Dual-energy X-ray absorptiometry (DXA) (6). Currently, the reference standard, DXA, is favored for its reproducibility and low radiation exposure (7, 8). However, DXA measurements may be affected by technical and procedural factors, including patient positioning and analysis protocols, and its availability is largely restricted to hospital-based settings (8).

A significant limitation of DXA is its reliance on two-dimensional areal density, which does not account for bone volume. Consequently, larger bones may yield artificially inflated BMD values compared to smaller bones with identical volumetric densities, potentially leading to diagnostic inaccuracies based solely on bone size (9). Furthermore, it is largely confined to hospitals and remains poorly suited for bedside screening or use in rural and community settings. Quantitative ultrasound (QUS) has emerged as a viable non-invasive, portable, and radiation-free alternative, to DXA offering the portability required for broader clinical and community-based applications. Its parameters include broadband ultrasound attenuation (BUA), speed of sound (SOS), and stiffness index (SI) (10). QUS is usually used for easily accessible bones, such as the calcaneus, tibia, patella, metatarsal bones, phalanges, and radius (11). Large population-based datasets, including the UK Biobank, have further supported the use of QUS-derived estimates of bone status in epidemiological and genetic studies (12). Both DXA and QUS are associated with fracture risk prediction, particularly for hip fractures in older populations. However, established clinical guidelines emphasize that QUS should serve as a pre-screening tool to identify individuals who may benefit from confirmatory DXA assessment, rather than as a replacement for the reference standard (13, 14).

Although QUS has been expanded for clinical adoption, its performance relative to DXA remains inconsistent across different populations and devices. These discrepancies in the literature are often rooted in the heterogeneity of QUS technologies, differing skeletal measurement sites, and varying definitions of outcomes. Beyond fracture risk prediction, the degree of clinical interchangeability between these modalities remains contentious. This systematic review investigates these correlations to clarify the clinical utility of QUS and to define its specific role within established osteoporosis screening frameworks.

## 2 Methodology

This systematic review was conducted in accordance with the Preferred Reporting Items for Systematic Reviews and Meta-Analyses (PRISMA) guidelines, with a completed PRISMA checklist provided as a Supplementary File (15). The study protocol was registered in the International Prospective Register of Systematic Reviews (PROSPERO) under the registration number CRD420251146250 on 12 September 2025. The registration process was retrospective. No amendments were made to the protocol after registration.

### 2.1 Search strategy

For the selection of studies, the PICO framework was used: P (Population): Patents scanned for bone density measurement, I (Intervention): QUS used for scanning, C (Comparator): DXA used for comparison, O (Outcomes): Bone density measurements, including BMD, which reflects the mineral concentration/unit area, BMC quantifies the total mineral amount in a bone region, SOS reflects bone density and elasticity, BUA measures the reduction in the ultrasound signal when it passes through bone, SI is the combination of SOS and BUA, diagnostic accuracy and precision, correlation, and clinical outcomes. These parameters were selected because they represent both the structural and functional aspects of bone health. The literature search was conducted in PubMed, Scopus, Web of Science, EMBASE, Google Scholar, and Cochrane Library. The search covered publications from January 2005 to July 2025. The search terms were strategically combined using Boolean operators and included variations of the key concepts (**Table 1**).

**Table 1:** Search terms and their combinations to search relevant literature.

Category	Search terms
Primary measurement techniques	“quantitative ultrasound,” OR “QUS,” OR “dual-energy X-ray absorptiometry,” OR “DXA,” OR “DEXA”
Clinical parameters	“bone density,” OR “bone mineral density,” OR “BMD,” OR “osteoporosis diagnosis”
Study types	“comparative studies,” OR “validation studies,” OR “clinical trials”
Population terms	“postmenopausal,” OR “elderly,” OR “adult,” “children,” OR “diabetic”
Outcome	“correlation” OR “accuracy,” OR “precision,” OR “sensitivity,” “specificity”
Final Search String	(1 AND 2 AND 3 AND 4 AND 5)

### 2.2 Inclusion and exclusion criteria

Studies were eligible if they were comparative and analyzed the two modalities of bone density assessment. In terms of outcomes, the primary outcome was direct comparison of bone density measurements, with secondary outcomes including diagnostic accuracy and precision, correlation between techniques, and clinical outcomes, where possible with clear reporting around measuring protocols. Studies published between January 2005 and July 2025 in English language and peer-reviewed journals.

Studies that did not compare the two modalities with accuracy and precision outcomes were excluded. In addition, case reports, reviews, commentaries, animal studies, non-English publications, conference abstracts without full texts, letters to the editor, and opinion pieces were excluded. Exclusion criteria were also applied on technical grounds, including obsolete technology, non-standard protocols, lack of description of methodology, absence of statistical analysis, and insufficient quality control, among others.

### 2.3 Studies selection process

Studies were initially identified through searches of multiple databases, and duplicate records were excluded. The titles and abstracts of the retrieved records were screened by the author according to predefined inclusion and exclusion criteria. Full-text articles of potentially relevant studies were subsequently retrieved and assessed for eligibility by the author. Eligibility criteria were uniformly applied across all screening stages to ensure consistency in study selection. Studies excluded at the full-text stage were documented, along with the reasons for exclusion. All studies meeting the eligibility criteria were included in this systematic review for qualitative synthesis.

### 2.4 Data extraction process

Four areas were covered in the extraction process. Study characteristics were extracted first, including the author, year of publication, study design, methodology, sample size, population characteristics, study geographical location, and setting. Second, technical parameters were recorded, including the measurement protocols for each modality, equipment specifications and calibration methodologies, quality control procedures, and information on the measurement sites and parameters. Third, outcome data obtained (i.e., the number of participants meeting defined primary and secondary outcomes, estimates of statistical analyses performed, correlation coefficients, and diagnostic accuracy measures). Finally, quality indicators were evaluated to assess the strength of the study design, statistical handling, control of confounders, and completeness of follow-up.

### 2.5 Definition of study variables

The primary variables of interest included BMD measurements obtained using DXA at clinically relevant skeletal sites and QUS-derived parameters, including BUS, SOS, and SI, as defined in the original studies. The outcome variables included reported measures of association between QUS and DXA, diagnostic performance metrics where available, and fracture-related outcomes. The secondary variables comprised skeletal measurement sites, participant demographic characteristics, and technical characteristics of the measurement devices, as reported in the included studies.

### 2.6 Quality assessment

Standardized evaluation tools relevant to each study design were employed to appraise the methodological quality of the included studies. The quality of observational studies was evaluated using the Newcastle–Ottawa Scale according to selection, comparability, and outcome assessment. The methodological quality of the *in vitro* studies was assessed using the QUIN assessment tool. This assessment tool comprises items such as aim/objectives, sample size calculation, comparison group, methodology explanation, operator details, randomization, method of measurement of outcomes, outcome assessment or details, blinding, statistical analysis, and presentation of results. Single *in vitro* study was evaluated according to these items and rated as yes (allocating 1–2 points), no with 0 points or not applicable, high risk of bias (RoB) scores <50%, 50%–70% were the medium RoB, and >70% were the low RoB (16). These established tools provide a rigorous and objective assessment of the methodological quality of all eligible studies.

### 2.7 Certainty of evidence

The Grading, Reporting, Assessment, Development, and Evaluation (GRADE) framework was used to assess the certainty of the evidence. Outcomes were rated in the domain of methodological limitations, indirectness, imprecision, inconsistency, and publication bias as low, high, or not serious.

### 2.8 Statistical analysis

Descriptive statistics were used to summarize the study characteristics, population demographics, measurement parameters, and reported outcomes. Given the substantial clinical and methodological heterogeneity across the included studies, no quantitative pooling or meta-analysis was performed. Instead, the findings were synthesized descriptively, with reported associations between QUS and DXA measurements, diagnostic performance metrics, and fracture-related outcomes summarized narratively, as presented in the original studies. Where applicable, results were described according to relevant study characteristics to facilitate a qualitative comparison.

## 3 Results

### 3.1 Study selection and characteristics

#### 3.1.1 Identification of the included studies

**Figure 1:**
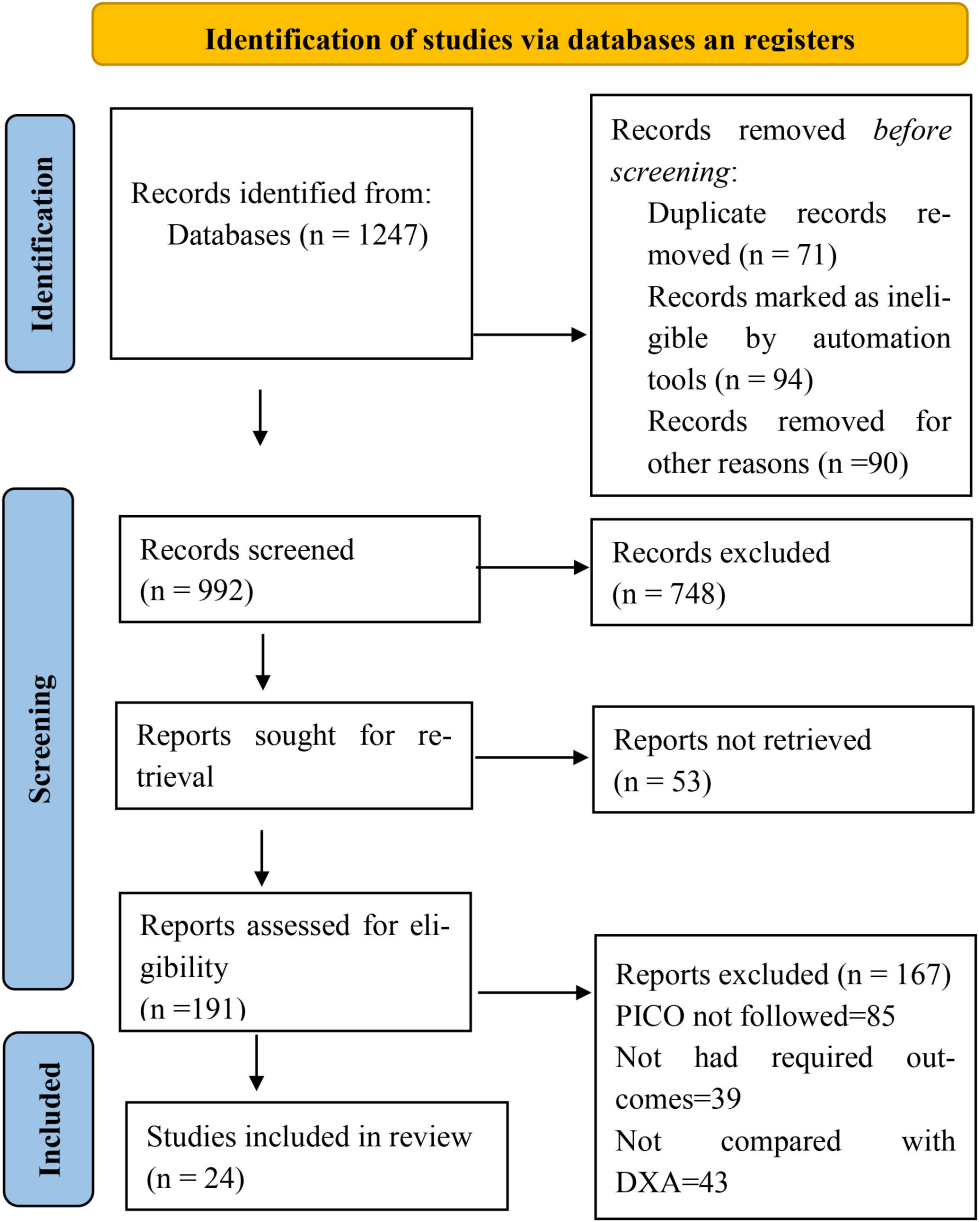
PRISMA flow chart showing study selection process.

A total of 1,247 records were identified through database searches. Prior to screening, 255 records were removed, including 71 duplicate records, 94 records marked as ineligible by automation tools, and 90 records removed for other reasons. The remaining 992 records were screened based on their titles and abstracts, of which 748 were excluded. Full-text reports were sought for 244 records, of which 53 were not retrieved. The remaining 191 full-text articles were assessed for eligibility, and 167 were excluded for predefined reasons (PICO not followed, n = 85; required outcomes not reported, n = 39; no comparison with DXA, n = 43). Ultimately, 24 studies were included in the qualitative synthesis (**Figure 1**).

#### 3.1.2 Demographic and study population characteristics

A total of 24 studies conducted across multiple countries were included, encompassing populations from Europe, Asia, Africa, and Oceania (**Table 2**). All studies followed non-randomized designs, with the majority employing cross-sectional methodologies (17–27), followed by retrospective (28–31), prospective (32, 33, 40), and longitudinal designs (34, 35). A single study followed the cohort, validation, *in vitro*, and case-control study designs (**Table 2**).

**Table 2:** Comprehensive narrative analysis (study and participants) of included studies.

Study characteristics					Participant characteristics			
Study ID	Country	Study design	Sample size	Study settings	Gender (M:F)	Age (Years)	BMI (Kg/m^2^)	Comorbidities
(19)	Saudi Arabia	Cross-sectional	437 (Osteoporosis, osteopenia, normal)	Primary care clinics, employee health clinic	0:437	47	NA	NA
(37)	Germany	*In vitro*	26 (Cadavers)	Hospital-based	12:14	56–96	NA	NA
(24)	China	Cross-sectional	Postmenopausal with T2DM:76, Postmenopausal without T2DM:86	Hospital-based	Postmenopausal with T2DM: 0:76, Postmenopausal without T2DM: 0:86	46–83	24.2	Diabetes
(22)	Greece	Cross-sectional	Severe hemophilia A: 17, moderate hemophilia: 10	Hospital-based	NA	11.75–13.19	NA	Hemophilia and one patient with hepatitis C
(27)	Sweden	Cross-sectional	80 postmenopausal women	Hospital-based	0:80	63	24.6	NA
(29)	India	Retrospective	101 (Normal, premenopausal, postmenopausal)	University campus	0:101	20–65	Normal = 23.47, premenopausal = 26.02, postmenopausal = 26.99	NA
(39)	Malaysia	Validation study	134 children	Part of SEANUTS	69:65	9.3	Male = 17.7, Female = 17.6	NA
(38)	Germany	Case-control	Postmenopausal women: 91, Health control: 91	Clinical	Postmenopausal women: 0:91, Health control: 0:91	Postmenopausal women: 78.2, Health control: 75.7	Postmenopausal women: 25.1, Health control: 26.6	NA
(30)	Thailand	Retrospective	181 children	School-based and part of SEANUTS	90:91	9.1	Male = 17.9, Female = 17	NA
(20)	Spain	Cross-sectional	107 adolescents	The PRO-BONE Study	107:0	13.2	18.7	NA
(28)	Nepal	Retrospective	115 (females with menopause)	Hospital-based	50:65	60.17	NA	Diabetes, COPD, hyperthyroidism, rheumatic arthritis, chronic renal failure
(25)	Ireland	Cross-sectional	56	Hospital-based	43: 13 postmenopausal	58	28.4	NA
(18)	New Zealand	Cross-sectional	124 children	Human nutrition research unit	58:66	10	18.7	NA
(34)	Vietnam	Longitudinal	2043	Part of the Vietnam Osteoporosis Study	773:1270	44–45.9	Male = 23, Female = 23.4	NA
(32)	Sweden	Prospective	62 diabetic patients	Foot clinics	34:28	50-65	T1DM = 24, T2DM = 27	Diabetes
(31)	Taiwan	Retrospective	772 osteoporotic and non-osteoporotic patients	Hospital-based	352:420	72.9	NA	NA
(35)	South Africa	Longitudinal	Child with HIV: 80, Child without HIV: 90	Hospital-based	Child with HIV: 40:40, Child without HIV: 51:39	7.14–7.29	16.1–16.6	HIV
(33)	China	Prospective	274 (Healthy, osteopenia, Osteoporosis)	Hospital-based	NA	53.78–67.11	Health = 24.40, Osteopenia = 23.87, Osteoporosis = 22.75	NA
(36)	UK	Cohort	216,753	UK-biobank	100,065:116,688	57.8–58.7	NA	NA
(21)	Thailand	Cross-sectional	67	Hospital-based	67:0	>50	NA	COPD
(26)	India	Cross-sectional	90 (postmenopausal women)	Hospital-based	0:90	55.82	25.40	Back pain
(40)	Switzerland	Prospective cohort	1345 (postmenopausal women)	Community-based (OsteoLaus cohort)	0:1345	65	NA	NA
(17)	Italy	Cross-sectional	201 (females were menopausal)	Two Italian centers	11:190	62.1	NA	Rheumatic musculoskeletal disease, CKD, and Diabetes
(23)	Uganda	Cross-sectional	167	Hospital-based and subtype of the CHAPAS-4 trial	85:82	9.4	NA	HIV

Age (years) is expressed as mean, SEANUTS, South East Asian Nutrition Surveys; M, Male; F, Female; BMI, Body Mass Index; CKD, Chronic Kidney Disease; COPD, Chronic Obstructive Pulmonary Disease; T1DM, Type 1 Diabetes Mellitus; T2DM, Type 2 Diabetes Mellitus; NA, Not Available.

The cumulative sample size across all 24 studies (223,676 participants), with individual study populations ranging from 27 to 216,753 participants (22, 36). Furthermore, 26 cadavers were used to compare the screening techniques (37). Most studies were hospital-based and conducted as part of larger research cohorts. Variation was also observed in the sex distribution of the included studies. Most studies (14, 58.3%) included mixed populations, while others specifically focused on menopause/postmenopausal females or males or targeted distinct groups such as diabetic patients or elderly individuals (19–21, 24, 25, 27, 29, 38). The mean age across studies ranged from 7.14 to 96 years (35, 37), and reported body mass index values ranged from 17 Kg/m^2^ to 28.44 Kg/m^2^ (25, 29). The comorbidities are listed in **Table 2**.

### 3.2 Technical characteristics of screening modalities

Overall, studies have compared DXA and QUS screening modalities and evaluated various bone parameters, including BMD, BMC, SOS, BAU, SI, and fracture rates. Various models of both screening modalities were used. The calibration methods also differed with some studies using manufacturer-supplied phantoms. Quality control procedures, including routine instrument checks, have been reported in some studies. A significant variation was observed in the measurement sites: QUS primarily targeted the calcaneus (heel), tibia, forearm, and radius, whereas DXA focused more on the lumbar spine, femoral neck, total hip, and sometimes the total body (**Table 3**).

**Table 3:** Technical characteristics of QUS and DXA in screening the participants.

Study ID	Technical characteristics					
	Modalities compared	Protocol measurements	Equipment specification	Calibration	Quality control procedures	Measurement sites
(19)	DXA vs QUS	DXA: BMD QUS: SOS, BUA	DXA: PIXI (Lunar GE, Radiation Corporation, Madison, WI USA) QUS: Hologic (Sahara clinical Bone Sonometer, USA)	References values were provided by manufacturer for comparison	Procedural manuals were used	DXA: Lumbar spine and femoral neck QUS: Heel
(37)	QUS vs DXA	DXA and QUS: Structural and mechanical properties of bone	QUS: DBM Sonic 1200 (IGEA, Carpi, Italy) DXA: QDR 1000 Hologic densitometer (Waltham, MA, USA)	NA	NA	Epiphyseal condyle site and meta-diaphyseal site
(24)	QUS vs DXA	QUS: SOS DXA: BMD	QUS: Sunlight Omnisense (7000P device) Petach Tikva, Israel DXA: DXA system (Lunar Prodigy, GE Healthcare, Madison WI)	NA	QUS: SOS verification phantom	QUS: Radius, phalanx, tibia DXA: Lumber spine, total hip, femoral neck
(22)	QUS vs DXA	QUS: SOS DXA: BMD	QUS: Sunlight Omnisense (7000P device) Petach Tikva, Israel DXA: Cronos bone densitometer (DMS, France)	QUS: Manufacturers’ verification phantom	NA	QUS: Peripheral bones DXA: Lumber spine
(27)	QUS vs DXA	QUS: SOS, BAU DXA: BMD, BMC	QUS: LUNAR (Achilles, Madison, WI, USA) DXA: LUNAR (DPX-L, Lunar Radiation Inc., Madison, WI, USA)	NA	Using a phantom every day	QUS: Calcaneal DXA: Lumber spine
(29)	QUS vs DXA	QUS: SOS DXA: BMD	QUS: Omnisense cbone densitometer DXA: Hologic DiscoverycWi	NA	NA	QUS: Radius and tibia DXA: Lumbar spine, total hip, and femoral neck
(39)	QUS vs DXA	QUS: SOS DXA: BMD	QUS: Commercial device (Omnisense 8000P, Sunlight, Petah Tikva, Israel) DXA: Hologic QDR Series Model Discovery W S/N 84687 (Hologic Inc., Waltham, MA, USA)	QUS: Verification phantom was used for calibration provided by the manufacturer DXA: Spine phantom was used for calibration, supplied by the manufacturer	NA	QUS: SOS DXA: BMD
(38)	DXA vs QUS	DXA: BMD QUS: SOS, BUA	DXA: Prodigy^®^ bone densitometer (GE/Lunar Corporation, Madison, WI, USA QUS: Achilles (Lunar, Madison, WI), Sahara (Hologic, Waltham, Massachusetts, USA), InSight (Achilles InSight, GE, USA), Omni (BeamMed, Sunlight), DBM Sonic Bone Profiler (Igea, Capri, Italy) and QUS-2 (Metra/Quidel Inc., San Diego, CA, USA) devices	NA	DXA: Quality assurance scans were performed on a daily basis QUS: As per manufacturer guidelines	DXA: lumbar spine (L1-L4) and the hip (femoral neck and total hip) QUS: Heel
(30)	QUS vs DXA	QUS: SOS DXA: BMD	QUS: Sunlight Omnisense, Petah Tikva, Israel (Model 8000P) DXA: Lunar Prodigy Pro, Madison, WI, USA (program encore 2008 Version 12.30)	NA	NA	QUS: Non-dominant arm DXA: Dominant forearm
(20)	DXA vs QUS	DXA: BMC QUS: SOS, BUA	DXA: GE Lunar Healthcare Corp, Madison, WI QUS: Lunar Achilles Insight (TM Insight; GE Healthcare, Milwaukee, WI)	Lumber spine phantom as recommended by the manufacturer for both devices	NA	DXA: Bilateral proximal femoral neck, and the total body QUS: Whole body
(28)	QUS vs DXA	QUS: SOS DXA: BMD	QUS: Sunlight MiniOmni bone sonometer (BeamMed Ltd., Tel Aviv, Israel)	DXA: Using spine phantom before the measurements	NA	Radius, left and right femur, spine
(25)	QUS vs DXA	QUS: SOS, BUA, SI DXA: BMD	QUS: General healthcare Lunar (Achilles InSight Densitometer DXA: Hologic Horizon scanner	QUS: Daily calibration DXA: International recommendations were used	DXA: International recommendations were used	QUS: Calcaneus DXA: Spine, lateral spine, total hip, femoral neck
(18)	QUS vs DXA	QUS: calcaneal BMD, SOS, BUA and SI DXA: TBLH, BMC, BMD, body composition	QUS: Sahara Clinical Bone Sonometre (Hologic Inc, USA) DXA: Hologic QDR Discovery A (Hologic Inc., Bedford, MA, USA) with APEX V. 3.2 software	DXA: Calibration materials were used	NA	QUS: Calcaneus DXA: Various sites
(34)	QUS vs DXA	QUS: BUA DXA: BMD	QUS: Portable ultrasound (Sahara, Hologic Corp., Bedford, MA, USA) DXA: Hologic Horizon densitometer (Hologic Corp., Bedford, MA, USA)	DXA: Phantom was used before measurement	NA	QUS: Calcaneus DXA: Femoral neck, total hip and lumbar spine
(32)	QUS vs DXA	Bone density and fractures	QUS: Lunar achilles bone densitometer DXA: Hologic ZDR-4500A	NA	NA	QUS: Calcaneus in both feet DXA: Spine and femoral neck
(31)	QUS vs DXA	DXA: BDM QUS: SOS	QUS: a Pegasus device (BeamMed Ltd., Tel Aviv, Israel)	DXA: A spine phantom was used prior to measurements	NA	Hip and spine
(35)	QUS vs DXA	QUS: SOS, BUA DXA: BMD	QUS: Lunar Achilles Insights device (GE Healthcare, Madison, WI, USA) DXA: Hologic Discover Wi bone Densitometer	NA	QUS: Built-in quality assurance test before each use	QUS: Calcaneal DXA: Lumber spine, whole body, radius
(33)	QUS vs DXA	DXA: BDM QUS: SOS	QUS: OSTEOKJ7000+ (Kejin, Nanjing, China) with a multichannel convolutional neural network processes the raw radiofrequency signal DXA: Prodigy (GE Healthcare, Waukesha, WI, USA)	NA	DXA: Daily standard procedures were followed	QUS: 1/3 distal radius of non-dominant hand DXA: femoral neck, hip and lumbar spine L1 L4
(36)	QUS vs DXA	QUS: SOS, BUA DXA: BMD	QUS: Sahara Clinical Sonometer (Hologic, Bedford, Massachusetts)	DXA: Manufacturer’s phantom was used	QUS: Using a phantom, as per manufacturer’s instructions DXA: Daily standard procedures were followed	QUS: Heel DXA: Total body, lumbar, femur
(21)	QUS vs DXA	QUS: SOS, BUA DXA: BMD	QUS: Acoustic Osteo-Screener ultrasound device (AOS-100, Aloka Co., Ltd., Japan) DXA: Osteosys DXAxum T, OsteoSys, Korea	NA	NA	QUS: Calcaneus DXA: Lumber spine, femoral neck
(26)	QUS vs DXA	BMD	NA	NA	NA	Neck, lumber, wrist
(40)	QUS vs DXA	QUS: SI, SOS, BUA DXA: BMD, TBS	QUS: Achilles Express (GE-Lunar, USA) DXA: Discovery A System (Hologic Inc., Waltham, MA, USA)	Daily calibration of QUS per manufacturer instructions	QUS and DXA performed by same operator; daily phantom calibration	QUS: Heel (right, or left if right fractured) DXA: Lumbar spine and hip
(17)	QUS vs DXA	QUS: SOS, BUA DXA: BMD	QUS: OsteoSys BeeTLe DXA: Lunar Prodigy (GE Healthcare, Madison, WI, USA) or Discovery Acclaim (Hologic, Waltham, MA, USA) devices	NA	All DXA scanners underwent daily quality control	Femoral, total hip and lumbar spine levels
(23)	DXA vs QUS	DXA: BMD QUS: SOS, BUA, BQI	DXA: DXA Hologic Discovery Wi DXA scanner Hologic Bedford Inc. Bedford MA, USA) QUS: SONOST 3000 (Osteosys, Seoul, South Korea)	DXA: The DXA scanner was calibrated daily using a spine phantom and auto air calibration for the whole body; QUS: The QUS machine was calibrated daily using a phantom prior to taking the measurements according to the manufacturer’s manual	NA	DXA: Head, lumbar spine; QUS: non-dominant foot QUS: Calcaneus

SI, Stiffness inDXA; TBLH, Total body Less Head; BMC, Bone Mineral Content; BMD, Bone Mineral Density; BUA, Broadband Ultrasound Attenuation; SOS, Speed of Sound; BQI, Bone Quality InDXA; DXA, Dual-Energy X-ray Absorptiometry; QUS, Quantitative Ultrasound.

### 3.3 Summary of key findings by modality comparison

Correlation analyses were the most frequently reported statistical approach used to evaluate the associations between DXA and QUS measurements. Reported correlation coefficients ranged from low (r = 0.17) to high (r = 0.86) (29, 31), with some studies reporting no statistically significant associations (22, 30). The diagnostic performance was evaluated in a subset of studies using receiver operating characteristic analysis. The area under the curve (AUC) values varied across studies, devices, and populations, as summarized in **Table 4**.

**Table 4:** Key findings of modalities used for screening of bone structures and quality.

Study ID	Outcomes				
	Statistical analysis performed	Correlation coefficient	Diagnostic accuracy	Follow-up	Conclusion
(19)	Correlation analysis	Low to moderate (r = 0.43–0.64, p = 0.000)	NA	NA	QUS may not be used as a screening tool
(37)	Correlation analysis	QUS significantly correlated with DXA (r = 0.69–0.79, p <0.05)	NA	NA	QUS is superior for structures
(24)	Correlation analysis	r = 0.26–0.75, p <0.05	NA	NA	Measurement did not change in parallel
(22)	Correlation analysis	No correlation	NA	NA	No correlation was observed
(27)	Correlation analysis and sensitivity	Significant correlation	Mean Sensitivity QUS: 79% Mean specificity QUS:45%	7 years	QUS is highly correlated with DXA
(29)	Correlation coefficient	Significant correlation (SOS radius and tibia (r = 0.858 and 0.860)	NA	NA	QUS is a sensitive screening tool
(39)	Correlation analysis, ROC	The mean difference between the two techniques was relatively large (0.6, p <0.001)	AUC: 0.94	NA	Radial QUS and DXA are not comparable
(38)	AUC	NA	DXA: AUC: Femoral neck = 0.69, Total hip = 0.71, Lumber spine = 0.59; QUS: Achilles = 0.68, Sahara = 0.63, InSight = 0.67, Omni = 0.60, DBM = 0.55 and QUS-2 = 0.51	NA	The Sahara, Achilles, and InSight QUS devices showed similar hip fracture discrimination when compared to DXA
(30)	Correlation	No correlation was observed	NA	NA	At radius, the SOS measurements were not appropriate for the assessment of bone quality status
(20)	Correlation	Fair to good intra-class correlation coefficients of agreement (r = 0.60–0.68) between DXA and QUS	NA	NA	QUS and DXA had comparable outcomes
(28)	Correlation, ROC analysis	Significant correlation	AUC = 0.69	NA	QUS is a sensitive screening tool
(25)	Correlation analysis, AUC	QUS significantly correlate	AUC = 0.77	NA	QUS identify patients with osteoporosis
(18)	Pearson correlation coefficients, linear regression	Positive correlations between QUS and DXA (r = 0.30–0.45, P <0.01)	NA	NA	Calcaneal QUS and DXA are not interchangeable methods for measuring bone density in children
(34)	Linear regression model	BUA modestly correlated with lumbar spine BMD (r = 0.34; P <0.0001) and femoral neck BMD (r = 0.35; P <0.0001)	NA	NA	QUS BUA is not a reliable method for screening osteoporosis
(32)	Correlation and logistic regression	Positive correlation	NA	10–11 years	QUS is an appropriate modality and is used for calcaneus as a fracture risk predictor
(31)	Correlation analysis, ROC analysis	Low correlation (r = 0.17)	AUC: 0.731	NA	A meaningful but low correlation between QUS and DXA
(35)	Correlation analysis	strong correlations at the calcaneus but modest at the radius	NA	12 months	The two methods did not correlate well longitudinally
(33)	AUC	NA	QUS: Sensitivity = 80.86%, Specificity = 84.23%, Accuracy = 83.05%	NA	QUS tools are promising future developmental directions
(36)	Reliability, correlation and sensitivity	Low to modest correlations (r = 0.29 to 0.44)	Sensitivity: Very poor (0.05–0.23) for osteoporosis, and poor (0.37–0.62) for osteopenia	NA	QUS has the potential to produce reliable absolute BMD measurements
(21)	Correlation, sensitivity, specificity	Significantly moderate correlation	Sensitivity = 10.4% Specificity = 94.7%	NA	QUS cannot replace DXA as an alternative
(26)	Correlation analysis, AUC	Significant correlation	Sensitivity = 86.36% Specificity = 86.76%	NA	QUS can be used as an alternative screening tool
(40)	Correlation and logistic regression analysis	Significant correlation	NA	6.7 years	QUS predicts fractures independently of FRAX, BMD, and TBS; suitable as pre-screening tool, not for monitoring
(17)	Repeatability, ROC analysis	NA	QUS: AUC: Femoral neck = 0.81, Total hip = 0.72, Lumber spine = 0.78	NA	QUS demonstrated good repeatability and performance similar to DXA
(23)	Correlation, AUC	Moderate to weak correlation	QUS was a weak predictor of DXA Z-score equal to or less than −2 (area under the ROC curve = 0.59)	NA	QUS may not be an appropriate substitute for DXA scan

BMD, Bone Mineral Density; BUA, Broadband Ultrasound Attenuation; SOS, Speed of Sound; DXA, Dual-Energy X-ray Absorptiometry; QUS, Quantitative Ultrasound; ROC, Receiver Operating Characteristic; AUC, Area Under the Curve; r, correlation coefficient.

### 3.4 Clinical applications and patient-specific outcomes

#### 3.4.1 Postmenopausal women

Eight studies included postmenopausal women (17, 24, 25, 27–29, 38, 40). These studies reported correlations between QUS parameters and DXA measurements, as well as sensitivity and repeatability metrics (**Table 4**).

#### 3.4.2 Elderly population

Studies conducted in elderly populations (50–96 years) have reported associations between QUS parameters and fracture-related outcomes, including hip fractures, using calcaneal measurements across multiple QUS devices (25–28, 33, 36, 38). The reported correlation coefficients varied across studies, with some demonstrating low correlations between QUS and DXA (r = 0.17) (31) (**Table 4**).

#### 3.4.3 Special populations

Studies involving children aged 7.1–13.2 years have reported correlations between QUS and DXA measurements ranging from no association to fair correlation (18, 22, 23, 30, 35, 39). However, one study reported a fair to good correlation (r = 0.60–0.80) between QUS and DXA measurements (20) (**Table 4**).

#### 3.4.4 Disease-specific analysis

Several studies have evaluated disease-specific populations, including patients with diabetes, COPD, rheumatic arthritis, and chronic renal failure. Reported AUC values ranged from 0.69 to 0.81 across different skeletal sites and outcomes (17, 28). However, other studies have found no comparable outcomes between QUS and DXA in these populations (21–24, 35) (**Table 4**).

### 3.5 Quality assessment outcomes

The methodological quality varied across the studies. Most studies demonstrated adequate reporting of the selection and comparability domains, although five studies lacked sufficient descriptions of participant selection (19, 22, 30, 36, 38). Adequate follow-up was reported in four studies (27, 32, 35, 38) (**Table 5**). The *in vitro* study demonstrated a low risk of bias across most assessed domains, although sample size justification, randomization, and blinding procedures were not reported (37).

**Table 5:** Methodological quality assessment.

Study ID	Study design	Selection				Comparability	Outcomes		
		Item 1	Item 2	Item 3	Item 4	Item 5	Item 6	Item 7	Item 8
(19)	Cross-sectional	No description	*	*	*	*	*	No follow-up	No statement
(24)	Cross-sectional	*	*	*	*	*	*	No follow-up	No statement
(22)	Cross-sectional	No description	*	*	*	*	*	No follow-up	No statement
(27)	Cross-sectional	*	No stated	*	*	*	*	*	*
(29)	Retrospective	*	*	*	*	*	*	No follow-up	No statement
(39)	Validation study	*	*	*	*	*	*	No follow-up	No statement
(38)	Case-control	No description	Not stated	*	*	*	*	*	*
(30)	Retrospective	No description	*	*	*	*	*	No follow-up	No statement
(20)	Cross-sectional	*	*	*	*	*	*	No follow-up	No statement
(28)	Retrospective	*	*	*	*	*	*	No follow-up	No statement
(25)	Cross-sectional	*	*	*	*	*	*	No follow-up	No statement
(18)	Cross-sectional	*	*	*	*	*	*	No follow-up	No statement
(34)	Longitudinal	*	*	*	*	*	*	No follow-up	No statement
(32)	Prospective	*	*	*	*	*	*	*	*
(31)	Retrospective	*	*	*	*	*	*	No follow-up	No statement
(35)	Longitudinal	*	*	*	*	*	*	*	*
(33)	Prospective	*	*	*	*	*	*	No follow-up	No statement
(36)	Cohort	No description						No follow-up	No statement
(21)	Cross-sectional	*	*	*	*	*	*	No follow-up	No statement
(26)	Cross-sectional	*	*	*	*	*	*	No follow-up	No statement
(40)	Prospective	*	*	*	*	*	*	*	No statement
(17)	Cross-sectional	*	*	*	*	*	*	No follow-up	No statement
(23)	Cross-sectional	*	*	*	*	*	*	No follow-up	No statement

Items explanation (Cohort studies): Item 1: Representativeness of participants; Item 2: Selection of non-exposed cohort; Item 3: Ascertainment of exposure; Item 4: Demonstration that outcome of interest was not present at start of study; Item 5: Comparability of cohorts; Item 6: Assessment of outcomes; Item 7: Was follow-up long enough; Item 8: Adequate of follow-up of cohorts. Items explanation (Case-control study): Item 1: Is the case definition adequate; Item 2: Representativeness of the cases; Item 3: Selection of controls; Item 4: Definition of controls; Item 5: Comparability of cases and controls; Item 6: Ascertainment of exposure; Item 7: Same method of ascertainment for cases and controls; Item 8: Non-response rate. The symbol (*) indicates that the study met the specified criterion for that particular item.

### 3.6 Certainty of evidence

Overall, most studies achieved moderate methodological quality based on the Newcastle-Ottawa Scale, with several cross-sectional and retrospective designs scoring lower in the selection and follow-up domains. Common limitations included incomplete description of participant selection, lack of follow-up, and insufficient reporting of confounder control. Despite these issues, the outcome assessments were generally well reported.

In the methodological limitation domain, no serious concerns were identified, as most studies demonstrated a low risk of bias. Similarly, indirectness was not considered serious because the studies provided sufficient details of patient characteristics, screening modalities, and clinical outcomes. Imprecision was also judged as not serious, as the majority of studies adequately explained patient selection, although five studies did not provide complete details of patient selection. Both positive and negative results were reported across studies; thus, publication bias was not suspected. According to GRADE (**Table 6**), the overall certainty of evidence was rated as low to moderate, with inconsistency in correlation values remaining the main factor limiting confidence in the findings.

**Table 6:** Summary of certainty of evidence using the GRADE framework.

GRADE domain	Judgement description	Concerns
Methodological limitations	Overall, studies were found with low risk of bias, however, concerns in the selection of patients and follow-up exist (**Table 5**).	Not serious
Indirectness	Studies provided details of the characteristics of patients, screening modalities, and clinical outcomes (correlation coefficient and ROC).	Not serious
Imprecision	Overall, acceptable number of patients were included in the selected studies. Most studies clearly define the selection criteria for patients.	Not serious
Inconsistency	Low to fair correlation was reported.	Not serious
Publication bias	Even the funnel plot was not constructed for publication bias, however, we did not find any publication bias, as both negative and positive outcomes were reported.	Not suspected

ROC, Receiver Operating Characteristics.

## 4 Discussion

In this systematic review, QUS demonstrated a low-to-fair correlation with DXA across diverse populations, accompanied by variability in diagnostic accuracy and methodological quality among studies. These findings highlight both the potential and limitations of QUS when considered alongside DXA, particularly in the context of screening rather than diagnosis. The observed heterogeneity across studies underscores the importance of population characteristics, measurement sites, and device-specific factors when interpreting QUS performance.

Our findings are partially consistent with those of Flöter et al. (41), who reported the variable diagnostic performance and moderate sensitivity of calcaneal QUS compared with DXA. However, their review was limited to the calcaneus, whereas our analysis included multiple skeletal sites, offering a broader and more comprehensive evaluation of the skeletal sites. Similarly, a meta-analysis by Moayyeri et al. (42), demonstrated a significant association between heel QUS parameters and fracture risk, although the predictive accuracy was moderate and varied across studies. Importantly, their work focused mainly on fracture prediction rather than diagnostic agreement with DXA, highlighting the complementary role of QUS in relation to DXA.

Evidence from the included studies involving postmenopausal women and older adults suggests that QUS is associated with moderately correlated with DXA and has reported sensitivity values for the detection of low bone density. These findings support the potential role of QUS as a preliminary screening tool in high-risk populations, particularly in settings where access to DXA is limited. Early identification of bone loss in postmenopausal women is clinically relevant because therapeutic interventions become less effective with advancing age (43). In elderly populations, QUS might help triage individuals who require further DXA evaluation, especially in primary care and low-resource environments (44, 45).

In pediatric populations, the available evidence indicates a poor agreement between QUS and DXA measurements. Studies investigating children and adolescents have consistently shown weak or inconsistent correlations, suggesting that QUS cannot reliably substitute DXA in younger age groups (46, 47). Factors such as rapid skeletal growth, anatomical variability, and challenges in standardized measurement sites likely contribute to these findings, thereby limiting the clinical applicability of QUS in pediatric settings.

The findings were particularly inconsistent in patients with chronic conditions, such as diabetes, COPD, and chronic kidney disease. These populations exhibited variable correlations and diagnostic agreement between QUS and DXA, influenced by disease-related alterations in bone metabolism, measurement site selection, and T-score thresholds (14, 48, 49). These findings indicate that QUS should be used cautiously in clinically complex populations and highlight the need for population-specific calibrations and validations.

Substantial variability in the reported correlations across studies may be attributed to differences in QUS device models, manufacturers, calibration methods, skeletal measurement sites, and operator-dependent factors. These methodological differences limit the comparability across studies and complicate the interpretation of pooled findings.

From a clinical and public health perspective, the findings support the use of QUS as a screening or triage tool rather than a diagnostic replacement for DXA. Its portability, affordability, and lack of ionizing radiation make it particularly attractive for large-scale screening in elderly and postmenopausal populations, especially in low-resource settings. However, confirmatory DXA assessment remains essential for diagnosis and clinical decision-making.

This review provides a comprehensive synthesis of the evidence comparing QUS and DXA across multiple populations and skeletal sites. The strengths of this review include the broad inclusion of study designs and populations and the application of standardized quality and certainty assessment tools. Limitations include the inability to perform a meta-analysis due to substantial heterogeneity, lack of standardized QUS protocols, and variability in outcome definitions and thresholds. Additionally, the overall certainty of the evidence was rated as low to moderate, primarily due to inconsistency and indirectness.

Future studies should focus on standardizing the QUS measurement protocols, device calibration, and diagnostic thresholds. Prospective studies with consistent outcome reporting and longer follow-up are required to better define the role of QUS in fracture risk assessment and screening. Population-specific validation studies, particularly in pediatric and disease-specific cohorts, are essential for improving clinical confidence and applicability.

## 5 Conclusion

This systematic review summarizes the available evidence comparing QUS and DXA for bone assessment in various populations. The findings indicate that QUS may serve as a preliminary screening tool, particularly in older adults and selected clinical populations, although considerable variability exists across studies. Importantly, the current findings do not support the use of QUS as a substitute for DXA, which remains the diagnostic reference standard for osteoporosis. Further well-designed studies with longer follow-ups and standardized methodologies are required to better define the role of QUS in osteoporosis screening pathways.

## Data Availability

Publicly available datasets were analyzed in this study. All data extracted for this review are available from the corresponding author upon reasonable request. No analytical code was generated for this review. All materials used during the review process, including data extraction forms and screening templates, are available upon request.
